# Association Between *ACE* (I/D) Polymorphism and Essential Hypertension (EH): An Updated Systematic Review and Meta-Analysis

**DOI:** 10.3390/ijerph23030397

**Published:** 2026-03-20

**Authors:** Athina Smallwood, Elizabeth Akam, David John Hunter, Monica Singh, Puneetpal Singh, Sarabjit Mastana

**Affiliations:** 1 School of Sport, Exercise and Health Sciences, Loughborough University, Loughborough LE11 3TU, UK; anz23umu@uea.ac.uk (A.S.); e.c.akam@lboro.ac.uk (E.A.); d.j.hunter@lboro.ac.uk (D.J.H.); 2 Department of Human Genetics, Punjabi University, Patiala 147002, India; singhmonica2017@gmail.com (M.S.); puneetpalsingh@pbi.ac.in (P.S.)

**Keywords:** *ACE* (I/D), essential hypertension, Indian, African, European, Middle Eastern, Hispanic, Chinese

## Abstract

**Highlights:**

**Public health relevance—How does this work relate to a public health issue?**

- Essential hypertension (EH) is a major global contributor to morbidity and mortality and is a leading risk factor for cardiovascular diseases (CVDs) such as stroke, heart disease, and heart failure.
- Understanding population-specific genetic risk factors is directly relevant to public health because hypertension prevalence varies across ethnic groups, and early detection and prevention strategies depend on accurate risk stratification.

**Public health significance—Why is this work of significance to public health?**

- The meta-analysis identifies a significant association between the *ACE* D allele and increased risk of EH in several populations (Indian, European, Chinese), highlighting the genetic component in hypertension and its interplay with environmental drivers.
- Since EH is highly prevalent and modifiable, understanding contributing genetic factors can enhance targeted intervention strategies, potentially reducing the burden of CVDs globally.

**Public health implications—What are the key implications or messages for practitioners, policy makers and/or researchers in public health?**

- For practitioners and researchers: Knowledge of genetic risk may guide personalised risk assessment and preventive counselling, particularly in populations in which stronger associations are observed.
- For policymakers: Findings underscore the need for ethnically tailored public health strategies, including hypertension screening guidelines, culturally and genetically informed prevention programs, and investment in genomic public health infrastructure.

**Abstract:**

Background: Essential hypertension (EH) refers to elevated arterial blood pressure with unknown etiology, which becomes more prevalent with age. Although the D allele of the *ACE* (I/D) polymorphism has been linked to EH, this association is not consistent across global populations. This systematic review and meta-analysis examined the relationship between the *ACE* (I/D) polymorphism and EH in diverse populations to determine the comparability of effect sizes and explore potential implications for public health planning. Methods: Case–control and cohort studies published in the last 20 years were reviewed from the main databases (PubMed, Scopus and Embase) using specific inclusion and exclusion criteria. Genotype data were used in meta-analyses using different genetic models. Results: Twenty-two studies with 7690 participants (3886 cases and 3804 controls) were included. Significant associations were observed between the *ACE* D allele and EH across allelic (OR = 1.37, 95% CI: 1.14–1.63), recessive (OR = 1.61, 95% CI: 1.21–2.13), dominant (OR = 1.37, 95% CI: 1.13–1.67), and homozygote (OR = 1.79, 95% CI: 1.31–2.45) models. Subgroup analyses showed significant associations in Indian and European populations, while African, Middle Eastern and Hispanic groups showed no statistically significant associations. Conclusions: The findings support a significant association between the *ACE* D allele and EH in several populations, though associations vary by ethnicity.

## 1. Introduction

Essential hypertension (EH) is characterised by chronically elevated arterial blood pressure without an identifiable secondary cause [1]. EH accounts for 90–95% of all cases of hypertension and remains a major contributor to global morbidity and mortality [2]. EH prevalence rises markedly with age and displays clear sex and ethnic disparities [3,4,5]. As a major modifiable risk factor for cardiovascular diseases (CVDs), EH substantially increases the risk of coronary artery disease, stroke, heart failure and premature death [6,7,8,9,10]. EH arises from a multifactorial interplay of genetic, epigenetic, and modifiable/non-modifiable environmental factors. Established non-modifiable risk factors include advancing age, family history of EH, and comorbidities such as diabetes and chronic kidney disease. In contrast, modifiable risk factors include unhealthy diet, physical inactivity, tobacco smoking, alcohol consumption, type 2 diabetes (T2D) and obesity [5,6,8]. Genetic factors are estimated to account for 30–60% of inter-individual variation in blood pressure [11,12], with particular emphasis on pathways regulating vascular tone and fluid balance. Among these, the renin–angiotensin–aldosterone system (RAAS) plays a central role in blood pressure homeostasis and is pivotal to the pathogenesis of EH [13,14]. Research indicates strong links between genetic variations of the RAAS encoding genes and both the genetic basis of EH and antihypertensive treatment, particularly associating increased risk with angiotensinogen (*AGT*), angiotensin-II receptor 1 (*AGTR1*) and angiotensin-converting enzyme (*ACE*) [13] genes.

ACE, predominantly expressed in the vascular endothelium of the lungs and kidneys, is an important circulating enzyme in the RAAS that catalyses the conversion of angiotensin I to potent vasoconstrictor angiotensin II and degrades the vasodilator bradykinin [14,15]. Variation in ACE activity has direct implications for vascular resistance and blood pressure regulation. A commonly studied genetic variant influencing ACE activity is the *ACE* (I/D) polymorphism, characterised by the presence of an insertion (I) or deletion (D) allele of a 287 non-coding base pair *Alu* repeat sequence in intron 16 of the human *ACE* gene, which is located at chromosome 17q23 [16]. *ACE* (I/D) polymorphism is represented by multiple reference sequence numbers (rs1799752, rs4340, rs13447447 and rs4646994) and results in three genotypes (II, ID and DD). The *ACE* (I/D) polymorphism accounts for up to 50% of the variability in circulating ACE levels [17]. Individuals with the DD genotype consistently exhibit the highest plasma ACE concentrations; those with II show the lowest, and those with ID have intermediate levels, suggesting a link between the D allele and increased vasoconstriction and hypertension risk [18].

Despite this mechanistic understanding, epidemiological findings regarding the association between the *ACE* (I/D) polymorphism and EH have been inconsistent across populations. Some studies in European, Indian and Chinese cohorts report a significant association between the D allele and elevated blood pressure or increased risk of EH, whereas others fail to demonstrate such relationships. A 2021 systematic review and meta-analysis emphasised these discrepancies and highlighted the need for further population-specific analyses to determine whether the *ACE* (I/D) variant can serve as a clinically useful marker for risk prediction, diagnosis, or therapeutic stratification [9].

To address these inconsistencies and incorporate newly available evidence, this updated systematic review and meta-analysis aims to further evaluate the association between the *ACE* (I/D) polymorphism and EH across diverse population groups. By comparing effect sizes between populations, this study seeks to clarify the genetic contribution of the *ACE* locus to EH and explore its potential implications for precision medicine and public health planning. We hypothesise that the D allele is associated with higher systolic blood pressure, with the DD genotype conferring the greatest elevation compared with the ID and II genotypes.

This systematic review was intentionally scoped as a genetic association meta-analysis focused on the *ACE* (I/D) polymorphism, a sentinel RAAS variant with strong biological plausibility. Environmental moderators (e.g., diet, adiposity, alcohol and tobacco use, socioeconomic disadvantage, and air pollution) were not meta-analytically modeled because primary studies seldom reported these covariates in a harmonised way. Accordingly, the present work should be interpreted as quantifying the genotype–phenotype association (*ACE* (I/D)→EH) rather than as a comprehensive risk model of EH.

## 2. Materials and Methods

### 2.1. Search Strategy

To accurately retrieve studies that addressed the association between *ACE* (I/D) polymorphism and EH, the electronic databases PubMed, Scopus and Embase were searched until 31 March 2025. *ACE* (I/D) polymorphism is represented by multiple rs numbers (rs1799752, rs4340, rs13447447 and rs4646994); therefore, all rs numbers were searched. The primary focus was on the *ACE* (I/D) polymorphism, and all papers were cross-checked to ensure that the use of different rs numbers corresponded to this polymorphism to avoid errors in the data extraction and analysis.

Search strategies were built with Boolean operators (AND, OR, NOT) to identify relevant published studies with key terms “essential hypertension”, “elevated blood pressure”, “systolic blood pressure”, “angiotensin-converting enzyme, *ACE*”, “insertion/deletion, I/D”, “*ACE* I/D (specific rs number)” “polymorphism”, with related terms to broaden the search. All included studies were published in English within the last 20 years and had to have full-text availability. The detailed search strategies are outlined in Supplementary Table S1.

### 2.2. Study Selection Criteria

The study selection criteria for this review were constructed using the PICO approach (Population, Intervention, Comparison, Outcome) [19].

Population: Studies that compared hypertensive and control groups were included in this study. Hypertensive subjects were defined with a clinical diagnosis of EH based on WHO’s diagnostic criteria for stage 1 hypertension (≥140/90 mm Hg). Controls were identified as healthy, with no medical history of any disease, and blood pressure below 140/90 mm Hg.

Participants aged below 18 were excluded to prevent puberty and growth from being confounders. Blood pressure increases at an accelerated rate during puberty, and after the first year of life, normal blood pressure likely increases more during puberty than at any other time [20]. No restrictions were applied to sex, ethnicity, race or the geographical origins of the studies.

This review excluded studies that (a) did not meet the requirements stated above for EH, (b) studied participants aged below 18, (c) included patients with underlying disease, or (d) were animal studies.

Intervention: This study evaluated the association between *ACE* (I/D) and EH. Therefore, case–control or cohort studies were included in this review. Interventions that were deemed ineligible included (a) studies other than case–control/cohort ones, (b) studies focused on treatment of hypertension, (c) studies that measured effects of variants other than ACE (I/D), (d) studies that measured synergistic effects, or (e) studies that addressed medical conditions other than EH.

Comparison: This review compared hypertensives with different genotypes with the controls from the same ethnicity. Ethnicity-matching between controls and hypertensives was fundamental to prevent a skew in data based on population differences. Studies focusing on only hypertensive individuals without a control population were excluded.

Outcome: The primary outcome measure was based on the proposed hypotheses. Therefore, the incidence of the D allele was compared between cases and controls. Consequently, studies must have included distributions of all *ACE* (I/D) genotypes for cases and controls.

**Figure 1:**
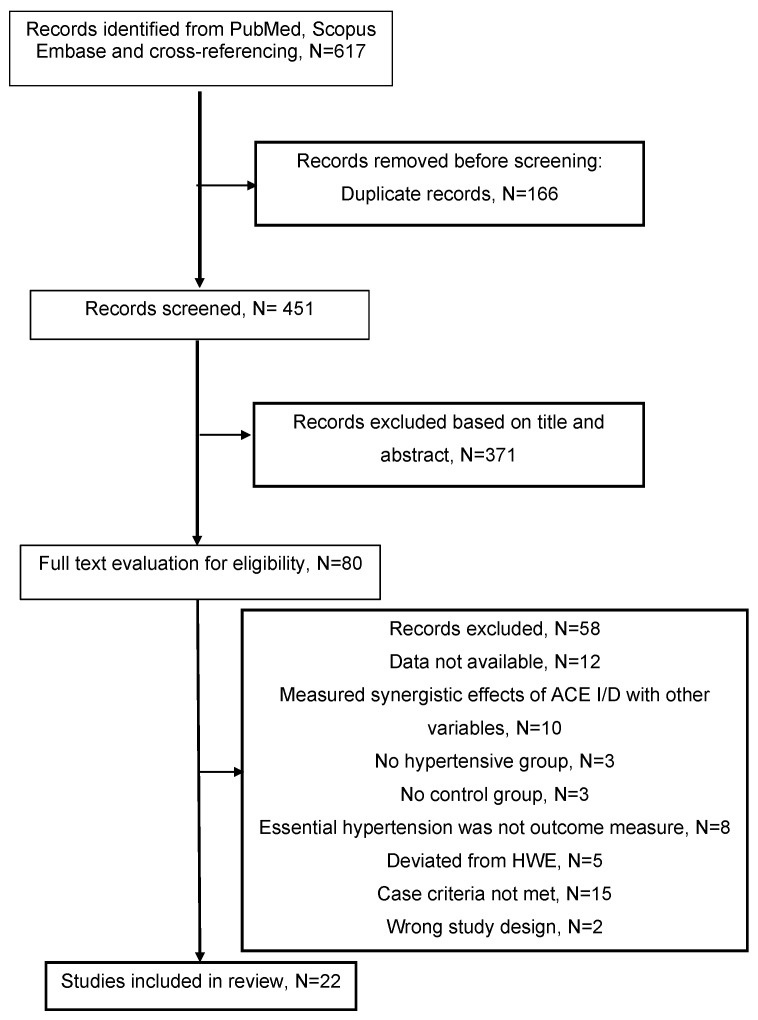
PRISMA flow diagram for this systematic review.

Selection process: The searches and selection of relevant articles were completed by two reviewers (AS and SM). Figure 1 shows that 617 articles were identified for screening following the initial search, and 166 duplicates were removed before title/abstract evaluation. In total, 371 articles with irrelevant titles and abstracts were excluded. The remaining 80 articles were screened for full-text evaluation. Following full-text evaluation, 22 studies were included in the final analysis.

Data extraction: Data were initially collected and recorded from the included studies by AS and reviewed independently by SM. Any discrepancies between reviewers were resolved by re-reviewing, discussion between reviewers and mutual agreement to include/exclude the studies. In some cases, an independent review was sought from other co-authors to resolve any differences. At each stage of the review, Covidence (http://www.covidence.org) was used to manage all data. The included studies meeting the criteria are tabulated in tables (Table 1, Table 2 and Table 3).

Quality assessment: Quality assessment of studies included in the review was completed using the Newcastle–Ottawa scale (NOS) to evaluate risk of bias specifically for case–control and cohort studies [21]. Each study was checked individually. Studies meeting the NOS criteria were marked with a star. Higher scores indicate a lower risk of bias. Studies scoring less than 6 stars were considered of questionable/low quality. Overall, the papers included scored highly on the NOS, with scores ranging between 6 and 8 stars. See Table 1 for the full quality assessment of papers.

**Table 1:** Quality assessment of included studies.

Study Type and Lead Author								
Cohort	Representativeness of exposed cohort	Selection of non-exposed cohort	Ascertainment of exposure	Demonstration that outcome of interest was not present at start of study	Comparability of cohorts based on the design or analysis	Assessment of outcome	Was follow up long enough for outcomes to occur?	Adequacy of follow up of cohorts
Das, (2008) [22]	-	*	*	*	**	*	-	-
Case–control	Is the case definition adequate?	Representativeness of cases	Selection of controls	Definition of controls	Comparability of cases and controls based on design or analysis	Ascertainment of exposure	Same method of ascertainment for cases and controls	Non-response rate
Saab, (2011) [23]	*	-	*	*	**	*	*	*
Badaruddoza, (2009) [24]	*	-	*	*	**	*	*	*
Martinez Cantarin, (2010) [25]	*	-	*	*	**	*	*	*
Patnaik, (2014) [26]	*	-	-	*	**	*	*	*
AbdRaboh, (2012) [27]	*	-	-	*	**	*	*	*
Sousa, (2018) [28]	*	-	*	*	**	*	*	*
Hussein, (2018) [29]	*	-	*	*	**	*	*	*
Tchelougou, (2015) [30]	*	-	*	*	**	*	*	*
Choudhury, (2012) [31]	*	-	-	*	**	*	*	*
Jiang, (2009) [32]	*	-	*	*	**	*	*	*
Amrani, (2015) [33]	*	-	*	*	**	*	*	*
Oscanoa, (2020) [34]	*	-	-	*	**	*	*	*
Hadian, (2022) [35]	*	-	-	*	**	*	*	*
Dhanachandra Singh, (2014) [36]	*	-	*	*	**	*	*	*
Patel, (2022) [37]	-	-	*	*	**	*	*	*
Tsezou, (2008) [38]	*	-	*	*	**	*	*	*
Pacholczyk, (2011) [39]	*	-	*	-	**	*	*	*
Roger, (2018) [40]	*	-	*	*	**	*	*	*
Yang, (2015) [41]	*	-	*	*	**	*	*	*
Starkova, (2022) [42]	*	-	*	*	**	*	*	*
Isordia-Salas, (2023) [43]	*	-	*	*	**	*	*	*

A study can be awarded a maximum of one star for each numbered item within Selection (S) and Exposure (E)/Outcome (O). A maximum of two stars can be awarded for Comparability (C).

**Table 2:** Summary of characteristics of included studies.

Lead Author (Year)	Study Design	Ethnicity/ Country	Total *N*	Cases	Control	Results
Das, (2008) [22]	Cohort	Indian	350	*N* = 185, SBP ≥160 mm Hg and/or a DBP ≥ 90 mm Hg or using antihypertensives	*N* = 165, Normotensive	DD genotype significantly associated with EH (OR = 7.483, *p* = 0.0007) * in a selected sub-sample
Saab, (2011) [23]	C-C	Lebanese	270	*N* = 124, patients taking antihypertensive drugs and/or bp ≥ 140/90	*N* = 146, never treated with antihypertensive and BP below 140/90	Significant difference between both groups across genotypes (*p* < 0.05) *, but OR for DD not significant (*p* = 0.08)
Badaruddoza, (2009) [24]	C-C	Indian	100	*N* = 50, SBP >140 mm Hg accompanied by DBP > 90 mm Hg	*N* = 50, BP below 140/90, clinically healthy	The DD genotype is not associated with EH
Martinez Cantarin, (2010) [25]	C-C	African American	412	*N* = 173, average of 8 BP readings ≥140 mmHg SBP and/or ≥90 mmHg DBP by trained staff	*N* = 239, same as case group, but average SBP ≥130 mm Hg and DBP ≥ 80 mm Hg	No association, adjusted *p* = 0.25
Patnaik, (2014) [26]	C-C	Indian	520	*N* = 246, SBP ≥ 140 or current antihypertensive medication	*N* = 274, no history of hypertension	Significant association between DD and EH (*p* < 0.001) after adjustment for confounders
AbdRaboh, (2012) [27]	C-C	Egyptian	203	*N* = 110, SBP greater than 140 mmHg and DBP greater than 90 mmHg and taking at least 1 antihypertensive medication	*N* = 93, not on antihypertensives	Mild increase in risk for EH (OR = 1.2, *p* > 0.05)
Sousa, (2018) [28]	C-C	Portuguese	1712	*N* = 860, diagnosed with high BP (≥140/90) on at least 3 occasions, and/or on one antihypertensive for at least 3 months	*N* = 852, never treated, and presented with bp < 140/90 mm Hg	DD genotype significantly associated with high blood pressure under recessive (OR = 1.233, *p* = 0.032) * and multiplicative models (OR = 1.173, *p* = 0.025) *
Hussein, (2018) [29]	C-C	Babylon	221	*N* = 123, diagnosed by specialist physicians with EH	*N* = 98, apparently healthy according to specialist physicians	DD genotype allied with EH (OR = 2.288, *p* = 0.024) *
Tchelougou, (2015) [30]	C-C	Burkina Faso	406	*N* = 202, BP ≥ 140/90 mmHg	*N* = 204, no CVDs present	Strong association between the *ACE* (I/D) polymorphism and the development of hypertension (DD vs. ID+II OR = 3.62, *p* < 0.00001) *
Choudhury, (2012) [31]	C-C	Indian	182	*N* = 101, SBP >140 mm Hg and DBP > 90 mm Hg, according to JNC 7 criteria	*N* = 81, SBP < 140 mm Hg and DBP < 90 mm Hg. There was no associated illness in these subjects.	Significant association between DD genotype and hypertension (DD vs. II OR = 3.85, CI 1.66–8.93) * and (DD vs. ID OR = 4.32, CI = 2.11–8.84) *
Jiang, (2009) [32]	C-C	Chinese	455	*N* = 220, SBP ≥ 140 mm Hg and/or DBP ≥ 90 mm Hg)	*N* = 235, SBP < 140 mm Hg and DBP < 90 mm Hg and the absence of a history of hypertension	Dominant model associated with EH ID+DD vs. II (OR = 1.171; CI = 1.00–1.37) *
Amrani, (2015) [33]	C-C	Algerian	145	*N* = 75, diagnosed with EH and blood pressure ≥ 140/90	*N* = 70, normotensives	D allele significantly associated with EH (*p* = 0.0002) *
Oscanoa, (2020) [34]	C-C	Peruvian	104	*N* = 65, diagnosed with EH, verified with clinical history and antihypertensive treatment	*N* = 39, no clinical EH diagnosis and no history of taking antihypertensives	No significant association between DD vs. ID + II (OR = 0.56, *p* = 0.34) or II vs. DD + ID (OR = 0.95, *p* = 0.92) and EH
Hadian, (2022) [35]	C-C	Iran	206	*N* = 102, diagnosed with EH	*N* = 104, no history of EH and clinically healthy	Risk of HTN in individuals with the I allele is lower than in those with the D allele (OR = 0.54; *p* = 0.005) *
Dhanachandra Singh, (2014) [36]	C-C	Indian	422	*N* = 211, SBP of ≥140 mm Hg and/or DBP of ≥90 mm Hg or prior diagnosis of EH by a physician or current use of antihypertensive medication or history of EH	*N* = 211, SBP ≤ 120 mm Hg and DBP ≤ 80 mm Hg and no disease or medication	Dominant model associated with EH in males (OR = 0.401, *p* = 0.0009) * but protectively
Patel, (2022) [37]	C-C	Indian	571	*N* = 279, based on patient self-report of a prior physician diagnosis and use of antihypertensives for a minimum of one year and patients with at least one parent being hypertensive were selected	*N* = 292, randomly selected from outpatients, on routine health check-ups and not suffering from EH	Increased odds of EH with DD genotype (OR = 2.2068, *p* < 0.0001) *
Tsezou, (2008) [38]	C-C	Greek	498	N = 194, SBP ≤ 120 mm Hg and DBP ≤ 80 mm Hg	N = 304, normotensive individuals and SBP ≤ 120 mm Hg and DBP ≤ 80 mm Hg	No associations found after adjustment for confounders between DD and ID with EH
Pacholczyk, (2011) [39]	C-C	Polish	246	*N* = 144, SBP ≥ 140 mmHg and/or DBP ≥ 90 mmHg on at least 2 separate occasions or when they used antihypertensive agents	*N* = 102, classified as normotensive if they met the following criteria: (1) SBP was <140 mm Hg and/or DBP was <90 mmHg on at least 2 separate occasions; (2) no family history of hypertension; and (3) no current use of antihypertensive drugs.	DD genotype carriers had over 2 times higher risk of hypertension than subjects with ID and II genotype (OR = 2.20, CI = 1.19–4.07) * and DD vs. II (OR = 2.96, CI = 1.52–5.76) *
Roger, (2018) [40]	C-C	Gabonese	132	*N* = 95, EH is diagnosed, with SBP ≥ 140 mmHg and/or DBP ≥ 90 mmHg, or being on antihypertensive therapy	*N* = 37, blood pressure less than or equal to 140/90 mm Hg), with no family history of hypertension (no direct hypertensive relatives	No significant relationship. DD vs. ID+II (OR = 2.02, *p* = 0.075).
Yang, (2015) [41]	C-C	Chinese	429	*N* = 244, SBP ≥ 140 mm Hg or DBP ≥ 90 mm Hg, or use of antihypertensive medication during the previous 2 weeks	*N* = 185, SBP < 140 mm Hg and DBP < 90 mm Hg. No history of EH or other diseases	No significant association between DD and EH (OR = 1.12, 95% CI = 0.844–1.477)
Starkova, (2022) [42]	C-C	Russia	69	*N* = 35, diagnosed with EH as per ICD-10	*N* = 34, relatively healthy	D allele associated with EH (OR = 3.16, *p* = 0.030) *
Isordia-Salas, (2023) [43]	C-C	Mexican	432	*N* = 224, previously diagnosed with EH or treated with antihypertensives	*N* = 208, no history of hypertension	D allele associated with EH (OR = 1.4, *p* = 0.02) *

* Statistically significant, C-C = case–control, BP = blood pressure, SBP = systolic BP, DBP = diastolic BP, EH = essential hypertension, N = sample size.

**Table 3:** Case and control group genotype distribution and associated Hardy–Weinberg (HW) *p*-values.

Study	Ethnicity	Cases			Controls			Control HWE *p*-Value
		II	ID	DD	II	ID	DD	
Das, (2008) [22]	Indian	12	4	19	14	18	3	0.40
Saab, (2011) [23]	Middle Eastern	9	37	78	12	58	76	0.84
Badaruddoza, (2009) [24]	Indian	11	16	23	13	27	10	0.55
Martinez Cantarin, (2010) [25]	African	37	74	59	37	102	84	0.52
Patnaik, (2014) [26]	Indian	87	99	39	88	103	16	0.06
AbdRaboh, (2012) [27]	African	17	59	34	16	52	25	0.21
Sousa, (2018) [28]	European	110	368	382	128	389	335	0.39
Hussein, (2018) [29]	Middle Eastern	33	55	35	41	38	19	0.07
Tchelougou, (2015) [30]	African	27	104	73	10	57	135	0.22
Choudhury, (2012) [31]	Indian	17	31	53	21	43	17	0.56
Jiang, (2009) [32]	Chinese	83	108	29	110	112	13	0.02 *
Amrani, (2015) [33]	African	25	40	10	43	25	2	0.47
Oscanoa, (2020) [34]	Hispanic	6	28	31	19	14	6	0.23
Hadian, (2022) [35]	Middle Eastern	11	46	45	4	36	64	0.70
Dhanachandra Singh, (2014) [36]	Indian	40	88	83	51	93	67	0.10
Patel, (2022) [37]	Indian	46	116	117	61	159	72	0.12
Tsezou, (2008) [38]	European	20	90	80	52	132	116	0.18
Pacholczyk, (2011) [39]	European	28	72	44	32	53	17	0.53
Roger, (2018) [40]	African	7	33	55	3	19	15	0.37
Yang, (2015) [41]	Chinese	97	106	41	82	73	30	0.06
Starkova, (2022) [42]	European	7	18	10	15	10	9	0.02 *
Isordia-Salas, (2023) [43]	Hispanic	68	112	44	83	98	27	0.81

HWE = Hardy–Weinberg equilibrium. * Shows departure from HWE.

### 2.3. Statistical Analysis

Meta-regression analyses (e.g., *ACE* (I/D) × sodium intake, pollution, BMI, smoking, alcohol, socioeconomic indicators) were not undertaken due to missing, insufficient and inconsistent reporting of these covariates across eligible studies.

The meta-analysis was conducted using Metagenyo [44] due to its suitability for genetic association studies and its ability to handle the specific statistical demands of polymorphism-based analyses. Metagenyo provides an integrated platform designed explicitly for the meta-analyses of genetic epidemiology data, offering predefined models such as allele contrast, dominant, recessive, and genotype-specific comparisons. Subgroup analysis was performed based on ethnicity described in the reviewed studies. Studies were classified into African, Chinese, European, Hispanic, Indian, and Middle Eastern groups.

This review evaluated associations of *ACE* (I/D) polymorphism and EH in the allele contrast model (also known as the allelic model) (D vs. I), recessive model (DD vs. DI+II), dominant model (DD+DI vs. II), homozygote model (DD vs. II) and DD vs. DI model to identify which model provides the higher/lower odds ratios and associated significance. These models were chosen to capture different possible mechanisms through which the polymorphism may influence disease risk. No single model can fully describe all plausible patterns of genetic effect, so analysing several models provides a comprehensive and unbiased assessment. These models not only allow the identification of the most plausible mode of genetic action but also strengthen the meta-analysis by revealing how sensitive the pooled effect estimates are to different modeling assumptions. This approach enhances interpretability and ensures that potential associations are not overlooked due to rigid inheritance assumptions. Significance was assessed using odds ratios where values greater than 1, 95% confidence intervals not passing through 1, and *p*-value ≤ 0.05 were regarded statistically significant. The results are displayed using forest plots, funnel plots and subgroup analyses. Heterogeneity was examined using I^2^ to prevent the number of studies affecting the outcome of the analysis [45]. Deeks et al.’s [46] suggested boundaries were used to examine heterogeneity. To account for high heterogeneity, the random effects model was interpreted with I^2^ values > 50%, and the fixed effects model was interpreted with I^2^ < 50%. The risk of bias was assessed using funnel plots. Asymmetry was tested using Egger’s test, where a *p*-value < 0.05 indicated publication bias was present. Sensitivity analysis by omission of one study at a time was done to assess for instability and changes in the significance of the effect estimate.

## 3. Results

### 3.1. Study Characteristics

Table 2 lists the characteristics of the included studies meeting the inclusion and exclusion criteria. Twenty-two studies, including 7690 participants (3886 cases and 3804 controls) aged 18+ were included in the meta-analysis. Twenty-one studies were case–control and one study was cohort. Sample size of studies ranged from 69 to 1712. Ethnic subgrouping was based on the description of the population in the original paper and the availability of at least two studies from the same ethnicity/region.

**Figure 2:**
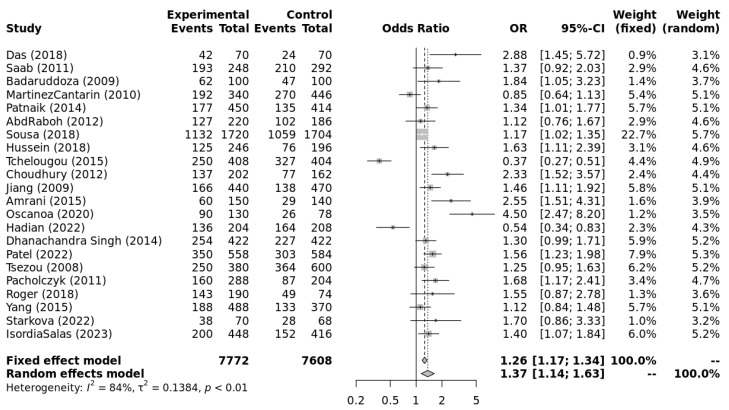
Forest plot of meta-analysis of the association between *ACE* (I/D) polymorphism and essential hypertension under the allele contrast model (D vs. I). Legend: Experimental: Hypertension Patients, Controls: healthy, Non-hypertensive controls, OR: odds ratio, CI: confidence interval, I^2^ = Heterogeneity, t^2^ = Between-Study Variance. References: Das [22], Saab [23], Badaruddoza [24], Martinez Cantarin [25], Patnaik [26], AbdRaboh [27], Sousa [28], Hussein [29], Tchelougou [30], Choudhury [31], Jiang [32], Amrani [33], Oscanoa [34], Hadian [35], Dhanachandra Singh [36], Patel [37], Tsezou [38], Pacholczyk [39], Roger [40], Yang [41], Starkova [42], Isordia-Salas [43].

**Figure 3:**
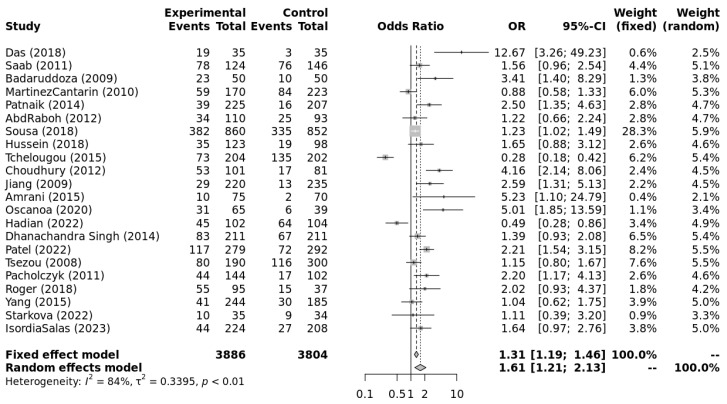
Forest plot of meta-analysis of the association between *ACE* (I/D) polymorphism with EH following the recessive model (DD vs. DI + II). Legend: Experimental: Hypertension Patients, Controls: healthy, Non-hypertensive controls, OR: odds ratio, CI: confidence interval, I^2^ = Heterogeneity, t^2^ = Between-Study Variance. References: Das [22], Saab [23], Badaruddoza [24], Martinez Cantarin [25], Patnaik [26], AbdRaboh [27], Sousa [28], Hussein [29], Tchelougou [30], Choudhury [31], Jiang [32], Amrani [33], Oscanoa [34], Hadian [35], Dhanachandra Singh [36], Patel [37], Tsezou [38], Pacholczyk [39], Roger [40], Yang [41], Starkova [42], Isordia-Salas [43].

**Figure 4:**
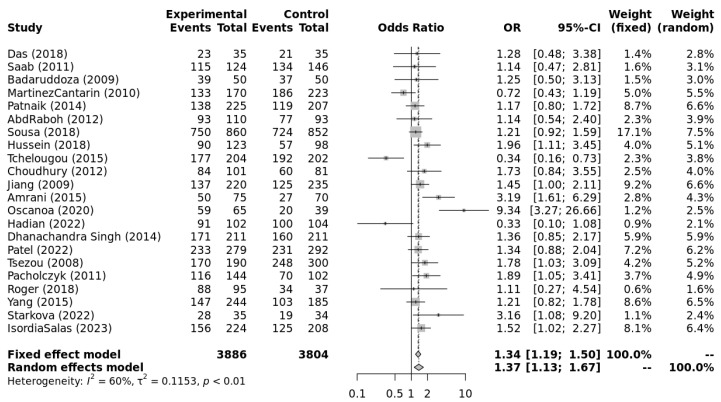
Forest plot of meta-analysis of the association between *ACE* I/D and EH under the dominant model (DD + DI vs. II). Legend: Experimental: Hypertension Patients, Controls: healthy, Non-hypertensive controls, OR: odds ratio, CI: confidence interval, I^2^ = Heterogeneity, t^2^ = Between-Study Variance. References: Das [22], Saab [23], Badaruddoza [24], Martinez Cantarin [25], Patnaik [26], AbdRaboh [27], Sousa [28], Hussein [29], Tchelougou [30], Choudhury [31], Jiang [32], Amrani [33], Oscanoa [34], Hadian [35], Dhanachandra Singh [36], Patel [37], Tsezou [38], Pacholczyk [39], Roger [40], Yang [41], Starkova [42], Isordia-Salas [43].

**Figure 5:**
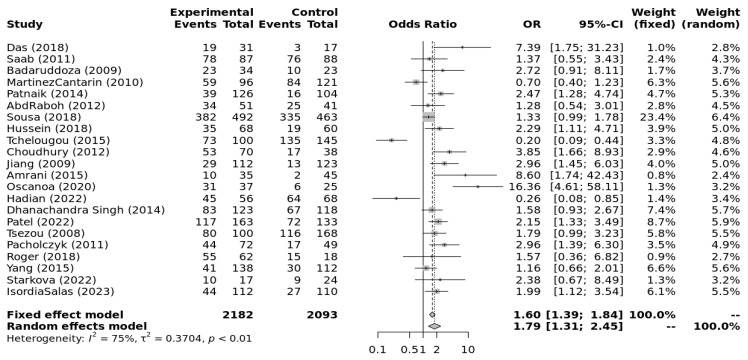
Forest plot of the association between *ACE* I/D and EH comparing homozygote genotypes (DD vs. II). Legend: Experimental: Hypertension Patients, Controls: healthy, Non-hypertensive controls, OR: odds ratio, CI: confidence interval, I^2^ = Heterogeneity, t^2^ = Between-Study Variance. References: Das [22], Saab [23], Badaruddoza [24], Martinez Cantarin [25], Patnaik [26], AbdRaboh [27], Sousa [28], Hussein [29], Tchelougou [30], Choudhury [31], Jiang [32], Amrani [33], Oscanoa [34], Hadian [35], Dhanachandra Singh [36], Patel [37], Tsezou [38], Pacholczyk [39], Roger [40], Yang [41], Starkova [42], Isordia-Salas [43].

**Figure 6:**
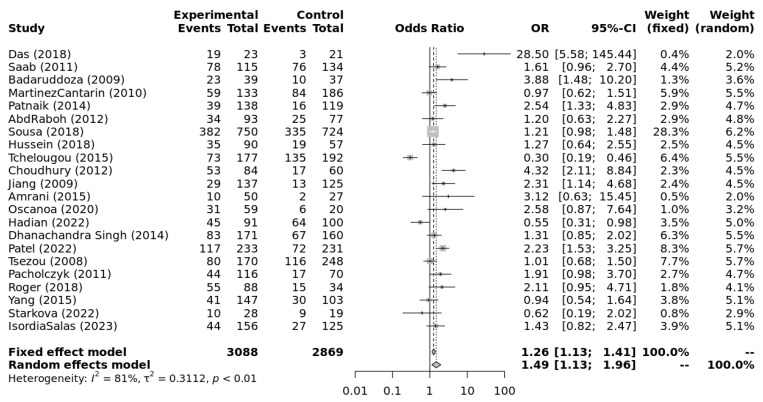
Forest plot of meta-analysis of the association between *ACE* (I/D) polymorphism and essential hypertension under the DD vs. DI model. Legend: Experimental: Hypertension Patients, Controls: healthy, Non-hypertensive controls, OR: odds ratio, CI: confidence interval, I^2^ = Heterogeneity, t^2^ = Between-Study Variance. References: Das [22], Saab [23], Badaruddoza [24], Martinez Cantarin [25], Patnaik [26], AbdRaboh [27], Sousa [28], Hussein [29], Tchelougou [30], Choudhury [31], Jiang [32], Amrani [33], Oscanoa [34], Hadian [35], Dhanachandra Singh [36], Patel [37], Tsezou [38], Pacholczyk [39], Roger [40], Yang [41], Starkova [42], Isordia-Salas [43].

The genotype data extracted from individual studies were input into Metagenyo [44] for processing and calculations (Table 3). Forest plots of meta-analyses under each model are presented in Figure 2, Figure 3, Figure 4, Figure 5 and Figure 6 and were interpreted using the random effects model, as substantial/considerable heterogeneity was observed [46]. There was no publication bias observed in any of the analyses as assessed by Egger’s test; all *p*-values were >0.05. Sensitivity analysis conducted by omitting one study at a time did not result in any significant changes in the significance level or odds ratio observed in any of the models.

### 3.2. Meta-Analysis

The meta-analysis was carried out using multiple models and at subgroup levels. The obtained results are presented using forest plots.

#### Meta-Analysis of EH in Different Genetic Models

Figure 2, Figure 3, Figure 4, Figure 5 and Figure 6 highlight the observed significant associations between the *ACE* (I/D) polymorphism and EH under different genetic models.

In subgroup analysis (Table 4), the allelic and dominant models showed significant associations for Chinese, European and Indian subgroups, while the recessive, homozygote and DD vs. DI comparisons were statistically significant only in European and Indian subgroups. Indian studies showed a higher overall effect as measured by odds ratios in most comparisons.

## 4. Discussion

Overall, this meta-analysis demonstrates that the *ACE* (I/D) polymorphism is significantly associated with EH across multiple genetic models, including allele contrast, dominant, recessive, homozygote, and DD vs. DI comparisons. Carriage of the D allele increased susceptibility to EH, with individuals carrying the DD genotype exhibiting the greatest risk. The relatively large combined sample size and balanced case–control distribution enhance the precision and reliability of the pooled estimates. All odds ratios were synthesised using random effects models to account for between-study heterogeneity [47], and no evidence of publication bias was detected in any model (*p* > 0.05). Nevertheless, caution is warranted because Hardy–Weinberg equilibrium (HWE) was violated in several control groups [32,42], potentially reflecting small sample sizes and/or genotyping errors.

Subgroup analysis revealed significant associations in Indian, European, and Chinese populations, whereas no associations were detected in African, Middle Eastern or Hispanic groups. Previous meta-analyses in Asian and European populations reported positive associations, consistent with our subgroup findings in Indian, European and Chinese cohorts [9,48]. Mengesha et al. [4] reported significant associations in African populations, whereas our analysis did not. Differences in the studies included, eligibility criteria, diagnostic definitions, and the proportion of hospital-based samples may partly explain these discrepancies, underscoring that methodological choices, not biological differences alone, can shape pooled estimates. Subgroup signals by ethnicity should be viewed as preliminary and hypothesis-generating. Differences likely reflect a combination of allele-frequency differences, study-design heterogeneity and unmeasured environmental modifiers rather than ethnicity per se.

Whether differences between ethnic groups would persist within the same country could not be tested here because few eligible studies enrolled multiple ethnicities under harmonised diagnostic criteria and exposure assessment. Even within a single nation, ancestry-informative genetic structure, epigenetic exposure, diet and sodium patterns, socioeconomic conditions, and environmental exposures (e.g., air pollution) vary across sub-populations and may modify ACE-related risk. Definitive resolution of these issues will require well-designed, multi-ethnic, within-country studies with standardised phenotyping and exposure capture.

A central observation in this study is pronounced ethnic variability in the *ACE* (I/D)-EH relationship. The meta-analysis design does not permit mechanistic interference, but several plausible explanations warrant consideration. First, allele-frequency differences can shift statistical power and influence vulnerability to confounding. Second, linkage disequilibrium patterns between *ACE* (I/D) and nearby functional variants may differ markedly across populations, potentially altering the biological relevance of the I/D polymorphism [17,49]. Third, gene–environment interactions, including diet, socioeconomic factors, and variable exposure to hypertension risk factors, may modulate the penetrance of genetic risk. These factors likely contribute collectively to the heterogeneous associations observed.

This study involves exclusive focus on EH, which provides a clear aim to this review, which is both a strength and a limitation, as it does not cover the broad spectrum of hypertension biology. All included studies scored highly on the NOS (6 *–8 *), supporting the validity of the extracted data. Additionally, the inclusion of studies published within 20 years of the last search date ensures all data extracted from each study is relevant by preventing outdated research methods from affecting the results. Limiting the design to case–control and cohort studies facilitated structured case–control comparisons, and controls were required to be normotensive without a history of hypertension.

Several limitations require acknowledgement. PICO search limitations narrowed the range of available studies for selection. Similarly, searching three main databases limited the studies accessible for review. Most included studies were from Indian, African or European cohorts, with limited representation from Middle Eastern, Hispanic and Australian populations, reducing generalisability. Across some studies, the diagnostic criteria for hypertension were inconsistent, and some case groups included individuals taking antihypertensive medications, whilst others excluded them. Many contributing studies relied on hospital-based controls, increasing susceptibility to selection bias. Potential population stratification was rarely addressed, particularly in multi-ethnic regions. Most published studies provided only crude genotype frequencies, restricting our ability to evaluate gene–environment interactions or perform adjusted meta-analysis. In particular, the absence of harmonised environmental and lifestyle data across studies precluded robust moderator analyses; our pooled estimates therefore isolate the genetic association (*ACE* (I/D)→EH) and should not be interpreted as environment-adjusted effects.

Despite these constraints, this meta-analysis supports the consensus that the *ACE* (I/D) polymorphism confers a moderate risk of EH in selected populations, which advances the concept of a modest genetic contribution to the development of EH that interacts with a multilayered network of physiological, environmental and possibly epigenetic influences. These findings reinforce the view of EH as a polygenic, multifactorial condition rather than one driven by a single variant.

Future work should prioritize (i) multi-ethnic, within-country cohorts with ancestry inference and harmonised covariates (dietary sodium, BMI, tobacco/alcohol, SES, air pollution), (ii) trans-ethnic meta-regression including design and exposure moderators, (iii) stratification by medication status and recruitment source (hospital vs. community), and (iv) interaction tests (e.g., *ACE* (I/D) × sodium intake; *ACE* (I/D) × pollution) and Mendelian randomisation where feasible.

## 5. Conclusions

This review provides updated evidence consistent with a modest association between the ACE D allele and increased susceptibility to essential hypertension, while acknowledging between-study heterogeneity; these findings support consideration of the D allele as a potential genetic risk factor and underscore the need for large, well-powered, population-focused studies.

## Figures and Tables

**Table 4:** Subgroup analysis regarding ethnicity to show associations between *ACE* (I/D) and essential hypertension compared with controls.

Allele/Genotype Model	Ethnicity	No. of Studies	Odds Ratio	95% CI	*p*-Value
D vs. I (allelic)	African	5	1.04	[0.56–1.92]	0.893
	Chinese	2	1.28	[1.04–1.56]	0.015 *
	European	4	1.23	[1.09–1.38]	<0.01 *
	Hispanic	2	2.43	[0.78–7.62]	0.127
	Indian	6	1.63	[1.33–1.99]	<0.01 *
	Middle Eastern	3	1.07	[0.56–2.05]	0.835
	All	22	1.37	[1.14–1.64]	<0.01 *
DD vs. DI+II (Recessive)	African	5	1.08	[0.467–2.48]	0.857
	Chinese	2	1.60	[0.66–3.89]	0.302
	European	4	1.27	[1.07–1.49]	0.005 *
	Hispanic	2	2.64	[0.89–7.79]	0.080
	Indian	6	2.74	[1.76–4.25]	<0.01 *
	Middle Eastern	3	1.08	[0.49–2.34]	0.842
	All	22	1.61	[1.21–2.13]	<0.01 *
DD+DI vs. II (Dominant)	African	5	0.99	[0.46–2.12]	0.979
	Chinese	2	1.33	[1.01–1.74]	0.039 *
	European	4	1.38	[1.09–1.73]	0.006 *
	Hispanic	2	3.53	[0.59–20.75]	0.163
	Indian	6	1.31	[1.05–1.63]	0.015 *
	Middle Eastern	3	1.02	[0.39–2.61]	0.976
	All	22	1.37	[1.13–1.67]	<0.01 *
DD vs. II (Homozygote)	African	5	1.02	[0.39–2.74]	0.972
	Chinese	2	1.80	[0.72–4.51]	0.211
	European	4	1.53	[1.19–1.96]	<0.01 *
	Hispanic	2	5.27	[0.67–41.34]	0.114
	Indian	6	2.29	[1.74–3.02]	<0.01 *
	Middle Eastern	3	1.01	[0.31–3.24]	0.986
	All	22	1.79	[1.31–2.45]	<0.01 *
DD vs. DI (Heterozygote)	African	5	1.04	[0.47–2.29]	0.924
	Chinese	2	1.44	[0.59–3.46]	0.420
	European	4	1.20	[1.01–1.43]	0.040 *
	Hispanic	2	1.61	[0.98–2.63]	0.057
	Indian	6	2.97	[1.75–5.05]	<0.01 *
	Middle Eastern	3	1.04	[0.53–2.04]	0.906
	All	22	1.49	[1.13–1.96]	<0.01

* Indicates significant.

## Data Availability

All data collected and used in the review are included in the relevant tables and figures. No new data was created.

## Supplementary Materials

The following supporting information can be downloaded at https://www.mdpi.com/article/10.3390/ijerph23030397/s1: Table S1: Search Strategy used for different databases; Table S2: PRISMA 2020 Main Checklist.
