# Supplementary material for: Association Between ACE (I/D) Polymorphism and Essential Hypertension (EH): An Updated Systematic Review and Meta-Analysis
Source: Int J Environ Res Public Health. 2026 Mar 20;23(3):397. doi: 10.3390/ijerph23030397 (PMC13027306; doi:10.3390/ijerph23030397)
Supplement: Supplementary file 1 [file ijerph-23-00397-s001.zip › ijerph-4153191-supplementary.pdf]

Supplementary Material

Scheme strategy—PubMed, Scopus and Embase were searched using the following searches and filters:

Table S1. Search Strategy used for different databases.

PICO Element	Search Terms
Population	Essential hypertension<br>Idiopathic hypertension<br>Primary hypertension<br>Hypertension<br>Elevated blood pressure<br>Raised blood pressure<br>Increased blood pressure<br>Systolic blood pressure
Intervention	ACE I/D polymorphism<br>Angiotensin converting enzyme<br>Angiotensin converting enzyme I/D polymorphism<br>ACE I/D polymorphism<br>ACE I/D polymorphism D allele<br>ACE I/D polymorphism DD genotype<br>Blood pressure<br>Renin-angiotensin system<br>Renin-angiotensin-aldosterone system<br>Normotensive control<br>Healthy control
Comparison	Normotensive control<br>Inactive control<br>Healthy control
Outcome	Increased D allele<br>Increased DD genotype

Search Terms	PubMed	Scopus	Embase
(ACE[Title/Abstract]) OR (angiotensin converting enzyme[Title/Abstract])	☒		
((insertion[Title/Abstract]) OR (deletion[Title/Abstract])) OR (I/D[Title/Abstract])	☒		
((polymorphism[Title/Abstract]) OR (variant[Title/Abstract])) OR (mutation[Title/Abstract])	☒		
(((hypertension[Title/Abstract]) OR (raised blood pressure[Title/Abstract])) OR (increased blood pressure[Title/Abstract])) OR (systolic blood pressure[Title/Abstract])	☒		
(((essential[Title/Abstract]) OR (primary[Title/Abstract])) OR (idiopathic[Title/Abstract])) AND (hypertension[Title/Abstract])	☒		
((((((ACE[Title/Abstract]) OR (angiotensin converting enzyme[Title/Abstract])) AND (I/D[Title/Abstract])) OR (insertion[Title/Abstract]))) OR (deletion[Title/Abstract])) AND (polymorphism[Title/Abstract])) OR (variant[Title/Abstract])	☒		
(((((essential hypertension[Title/Abstract]) OR (primary hypertension[Title/Abstract])) OR (idiopathic hypertension[Title/Abstract])) OR (increased blood pressure[Title/Abstract])) OR (raised blood pressure[Title/Abstract])) OR (systolic blood pressure[Title/Abstract])	☒		
(((((((ACE[Title/Abstract]) OR (angiotensin converting enzyme[Title/Abstract])) AND (I/D[Title/Abstract])) OR (insertion[Title/Abstract]))) OR (deletion[Title/Abstract])) AND (polymorphism[Title/Abstract])) OR (variant[Title/Abstract]) OR mutation[Title/Abstract]) AND ((((((essential hypertension[Title/Abstract]) OR (primary hypertension[Title/Abstract])) OR (idiopathic hypertension[Title/Abstract])) OR (increased blood pressure[Title/Abstract])) OR (raised blood pressure[Title/Abstract])) OR (systolic blood pressure[Title/Abstract]))	☒		
(((((((ACE[Title/Abstract]) OR (angiotensin converting enzyme[Title/Abstract]) OR (rs4646994[Title/Abstract]) AND (I/D[Title/Abstract])) OR (insertion[Title/Abstract])) OR (deletion[Title/Abstract])) AND (polymorphism[Title/Abstract])) OR (variant[Title/Abstract]) OR mutation[Title/Abstract]) AND ((((((essential hypertension[Title/Abstract]) OR (primary hypertension[Title/Abstract])) OR (idiopathic hypertension[Title/Abstract])) OR (increased blood	☒		

pressure[Title/Abstract])) OR (elevated blood pressure[Title/Abstract]) OR (raised blood pressure[Title/Abstract])) OR (systolic blood pressure[Title/Abstract]))		
TITLE-ABS (ACE OR {angiotensin converting enzyme})	☒	
TITLE-ABS(I/D OR insertion OR deletion)	☒	
TITLE-ABS(polymorphism OR variant OR mutation)	☒	
TITLE-ABS(hypertension OR {raised blood pressure} OR {increased blood pressure} OR {systolic blood pressure}	☒	
TITLE-ABS(essential OR primary OR idiopathic)	☒	
(TITLE-ABS(essential OR primary OR idiopathic)) AND (TITLE-ABS(hypertension OR {raised blood pressure} OR {increased blood pressure} OR {systolic blood pressure})) AND (TITLE-ABS(polymorphism OR variant OR mutation)) AND (TITLE-ABS(I/D OR insertion OR deletion)) AND (TITLE-ABS(ACE OR {angiotensin converting enzyme}))	☒	
(TITLE-ABS(essential OR primary OR idiopathic)) AND (TITLE-ABS(hypertension OR {raised blood pressure} OR {increased blood pressure} OR {elevated blood pressure} OR {systolic blood pressure})) AND (TITLE-ABS(polymorphism OR variant OR mutation)) AND (TITLE-ABS(I/D OR insertion OR deletion)) AND (TITLE-ABS(ACE OR rs4646994 OR {angiotensin converting enzyme}))	☒	
ace:ab,ti OR 'angiotensin converting enzyme':ab,ti		☒
'i/d':ab,ti OR insertion:ab,ti OR deletion:ab,ti		☒
polymorphism:ab,ti OR variant:ab,ti OR mutation:ab,ti		☒
(hypertension:ab,ti OR 'raised blood pressure':ab,ti OR 'increased blood pressure':ab,ti) AND 'systolic blood pressure':ab,ti		☒
(essential:ab,ti OR primary:ab,ti OR idiopathic:ab,ti)		☒
'ace':ab,ti OR 'angiotensin converting enzyme':ab,ti AND 'i/d':ab,ti OR insertion:ab,ti OR deletion:ab,ti AND polymorphism:ab,ti OR variant:ab,ti OR mutation:ab,ti AND (hypertension:ab,ti OR 'raised blood pressure':ab,ti OR 'increased blood pressure':ab,ti) AND 'systolic blood pressure':ab,ti AND (essential:ab,ti OR primary:ab,ti OR idiopathic:ab,ti)		☒
('i/d':ab,ti OR insertion:ab,ti OR deletion:ab,ti) AND (polymorphism:ab,ti OR variant:ab,ti OR mutation:ab,ti) AND (essential:ab,ti OR primary:ab,ti OR idiopathic:ab,ti) AND (ace:ab,ti OR 'angiotensin converting enzyme':ab,ti OR rs4646994:ab,ti) AND ('elevated blood pressure':ab,ti OR 'raised blood pressure':ab,ti OR 'increased blood pressure':ab,ti OR 'systolic blood pressure':ab,ti)		☒
((TITLE-ABS(insertion OR deletion OR I/D)) AND (TITLE-ABS({ACE enzyme} OR ACE OR {angiotensin converting enzyme}))) AND (TITLE-ABS(polymorphism OR variant OR mutation)) AND (TITLE-ABS(hypertension OR {systolic blood pressure} OR {high blood pressure}))	☒	
('i/d':ab,ti OR insertion:ab,ti OR deletion:ab,ti) AND ('ace enzyme':ab,ti OR ace:ab,ti OR 'angiotensin converting enzyme':ab,ti) AND (polymorphism:ab,ti OR mutation:ab,ti OR variant:ab,ti) AND (hypertension:ab,ti OR 'systolic blood pressure':ab,ti OR 'blood pressure':ab,ti)		☒
